# Undifferentiated high-grade pleomorphic sarcoma of the larynx treated with partial laringectomy^☆^

**DOI:** 10.1016/j.bjorl.2016.11.005

**Published:** 2016-12-23

**Authors:** Eduardo Cambruzzi, Ricardo Pedrini Cruz, Vinícius Grando Gava, Karla Lais Pêgas

**Affiliations:** a Universidade Federal do Rio Grande do Sul (UFRGS), Porto Alegre, RS, Brazil; b Universidade Luterana do Brasil (ULBRA), Porto Alegre, RS, Brazil; c Hospital Nossa Senhora da Conceição, Departamento de Patologia, Porto Alegre, RS, Brazil; d Santa Casa de Porto Alegre, Departamento de Patologia, Porto Alegre, RS, Brazil; e Hospital Nossa Senhora da Conceição, Departamento de Oncologia Cirúrgica, Porto Alegre, RS, Brazil

## Introduction

Malignant mesenchymal neoplasms affecting larynx are rare tumors. Undifferentiated high-grade Pleomorphic Sarcoma (UPS), formerly referred as malignant fibrous histiocytoma, is a high grade malignant neoplasm characterized by tumor cells with diffuse pleomorphism in the absence of a specific line of differentiation.^1^, ^2^, ^3^, ^4^ The process occurs more commonly in males and affects all age groups. Wide surgical margin is often indicated because of their high local recurrence rates. However, due to its rarity there are scarce studies with no statistic power to define the better outcome.^3^, ^5^, ^6^ Herein, the authors present a new case of undifferentiated high-grade pleomorphic sarcoma arising in larynx, and discuss pathological findings and surgical treatment of this rare tumor.

## Case report

**Figure 1:**
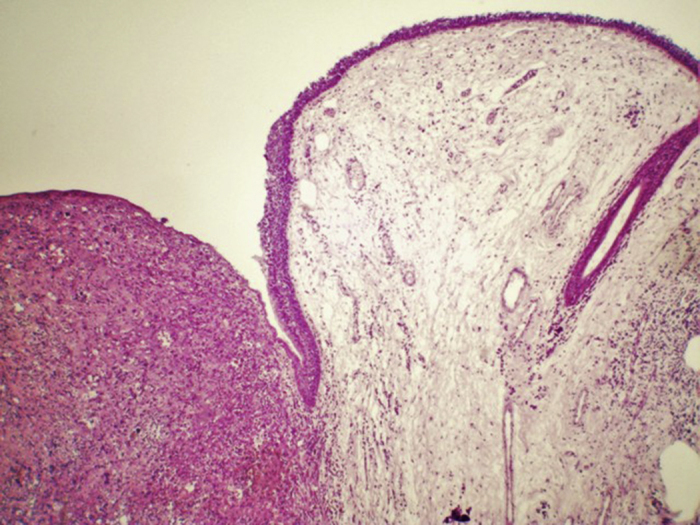
Undifferentiated high-grade pleomorphic sarcoma arising in larynx: A high-grade pleomorphic sarcoma affecting the lamina propria, surrounding by area of edema, Hematoxylin-eosin, 40×.

**Figure 2:**
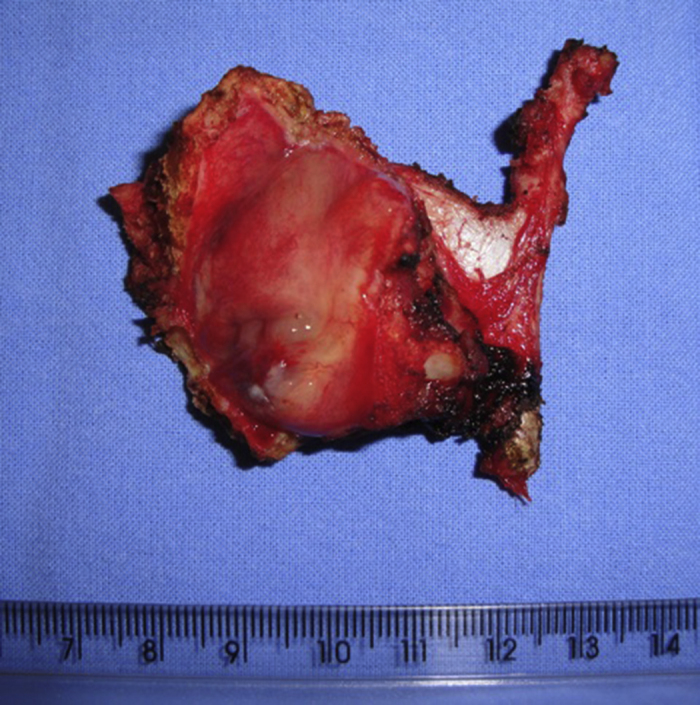
Undifferentiated high-grade pleomorphic sarcoma arising in larynx: A right frontolateral laryngectomy specimen.

**Figure 3:**
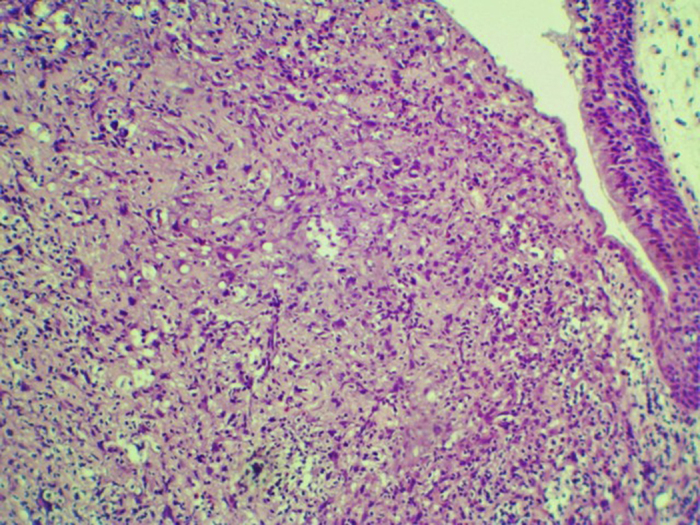
Undifferentiated high-grade pleomorphic sarcoma arising in larynx: A pleomorphic malignante neoplasia exhibiting epithelioid cells with marked nuclear and cellular atypia, Hematoxylin-eosin, 100×.

A 54 years male patient was admitted in hospital service due to clinical complaint of hoarseness in the last 9 months. Prior history included systemic hypertension and tobacco consumption. On physical examination and/or laryngoscopy, an elevated reddish brown lesion, with a central ulcerated area, compromising the right true vocal cord was determined. No signs of cervical lymphadenopathy were identified. The patient was submitted to laser cordectomy. On gross examination, the sample was composed by some gray, irregular fragments, weighting 1.0 g, with the largest fragment measuring 1.0 cm. On histological evaluation, a high grade pleomorphic malignant neoplasia, with spindle-cells arranged in a storiform pattern and a high mitotic index, was identified. The tumor was present in all fragments of the sample and compromised the radial margins. The process revealed positive immunoexpression for vimentin (diffuse) and smooth muscle actin (focal), and negative staining for specific muscle actin, calponin, AE1/AE3, S-100, desmin, h-caldesmon, CD31, CD34, and von Willebrand factor. The diagnosis of undifferentiated high-grade pleomorphic sarcoma of the larynx (storiform-pleomorphic malignant fibrous histiocytoma) was established (Fig. 1). The patient was then submitted to a right frontolateral laryngectomy (Fig. 2), without lymphadenectomy. Surgical specimen measured 5.5 × 4.0 × 3.0 cm, and showed a residual, soft, gray-brown nodule affecting the lamina propria of the right vocal cord (Fig. 3), which measured 0.9 × 0.6 × 0.5 cm. There was no evidence of vascular or lymphatic invasion. Surgical margins were free of neoplasia. After surgery patient had a salivary leakage, treated successfully with conservative management. After 5 years of follow up, the patient has no clinic or radiologic signs of recurrence.

## Discussion

Undifferentiated high-grade Pleomorphic Sarcoma (UPS), formerly referred as malignant fibrous histiocytoma, is a high grade malignant neoplasm characterized by tumor cells with diffuse pleomorphism in the absence of a specific line of differentiation. UPS manifests a broad range of histologic appearances, and most common patterns consists of a mixture of storiform and pleomorphic areas.^1^, ^2^, ^3^, ^4^ UPS arising in larynx is an uncommon tumor that characteristically compromises males (3:1) and occurs in all age groups (6–68 years). In larynx, the glottis is the site of predilection. Symptoms can vary to hoarseness, airway compromise, dysphagia or a sensation of foreign body in the throat.^3^, ^5^, ^6^ Usually, USP compromises somatic soft tissues as a slowing enlarging mass, and the thigh have been the most common location, followed by the upper extremity. Retroperitoneal UPS are related to anorexia, malaise, weight loss, and signs of increasing abdominal pressure. It is described association of UPS and previous radiation.^1^, ^2^, ^3^, ^6^, ^7^ Our patient, although, did not have exposure to radiation therapy.

On gross examination, UPS arising in larynx are usually a sessile to polypoid, firm; often ulcerate lesions, with a yellow-tan to gray-white cut surface. Some cases are found as a solitary, multilobulated, fleshy nodular mass that can measure until 10 cm in diameter. On cut surface, myxoid, hemorrhagic, and necrotic areas are common features.^3^, ^5^, ^8^, ^9^ On microscopic evaluation, UPS may be broadly divided into pleomorphic, spindle cell, round cell and epithelioid patterns, without an identifiable line of differentiation. Storiform areas consist of plump spindle cells arranged in short fascicles in a cartwheel, or storiform, pattern around slit-like vessels.^1^, ^3^, ^5^, ^8^, ^9^, ^10^ Pleomorphic areas contain plumper fibroblastic cells, rounded histiocyte-like cells arranged haphazardly with no particular orientation to vessels, and a large number of giant cells with multiple hyperchormatic irregular nuclei. Most tumors have a combination of storiform and pleomorphic areas, with preponderance on the latter, which also exhibit more accentuated pleomorphism and mitotic activity.^3^, ^5^, ^8^, ^9^, ^10^ Anaplastic tumor cells arranged haphazardly in sheets is a typical feature of USP. In general, the stroma consists of delicate collagen fibrils encircling individual cells. In some cases, collagen deposition is extensive and widely separates cells. Rarely, the stroma contains metaplastic osteoid or chondroid material.^1^, ^5^, ^8^, ^9^, ^10^ USP exhibiting numerous giant cells tends to be multinodular and composed of a mixture of spindle, rounded, and osteoclast-type giant cells. Some examples of USP have a proeminent xanthomatous and neutrophilic infiltrate, possibly related to elaboration of cytokines. USP can also exhibit myxoid and epithelioid elements.^1^, ^4^, ^5^, ^6^, ^7^, ^10^

The diagnosis of USP presupposes extensive sampling and evaluation of hematoxylin-eosin-stained sections, and immunohistochemistry technique is a fundamental tool to exclude other pleomorphic tumors. USP display features of fibroblasts/myofibroblasts, and can shows positive expression for smooth muscle actin. Focal expression for cytokeratins can be found. Stains for desmin and h-caldesmon are tipically negative.^3^, ^4^, ^6^, ^8^, ^9^ Differential diagnosis includes other malignant tumors that display a comparable degree of cellular pleomorphism. USP must be differentiated from sarcomatoid carcinoma, fibrosarcoma, myxofibrosarcoma, pleomorphic forms of liposarcoma, leiomyosarcoma, rhabdomyosarcoma, osteosarcoma, and chondrosarcoma.^3^, ^4^, ^6^, ^8^, ^9^

Squamous Cell Carcinoma (SCC) is the most common malignancy of the larynx, with the supraglotic and glottis regions being the most common locations.^3^, ^8^ SCC occurs mainly in adult males who abuse tobacco and alcohol. The tumor originates from the squamous mucosa or from ciliated respiratory epithelium that has undergone squamous metaplasia, or even from any grade of dysplasia arising in the epithelium. SCC may spread directly to contiguous structures, or via lymphatic and blood vessels to regional lymph nodes.^3^, ^8^, ^9^ Haematogenous metastases to more distant sites are unusual, and may occur in late stages of the disease. TNM staging, resection margins, proliferative index, and lymphovascular and perineural invasion are clinical predictive factors. Lymph node metastasis is the single most adverse prognostic factor in head and neck SCC.^3^, ^8^ This biological behavior of USP differs significantly from that related to SCC. USP is a mesenchymal high-grade malignant neoplasm originated from conjunctive tissue of the larynx. Haematogenous dissemination to the lungs is frequent, with no tendency to develop cervical metastases. Independent favorable prognostic factors related to disease-specific survival in USP are AJCC stage I or II, negative surgical margins, superficial location, myxoid subtype, and age less than 50 years. Wide surgical margins are usually indicated because of their high local recurrence rates, varying from 44% to 73%.^3^, ^5^, ^6^, ^8^, ^9^ It looks that radical resection of USP is a more efficacious method for improving survival and reducing recurrence. However, frontal partial laryngectomy and laryngomicrosurgery were previously described with good outcomes. Chemotherapy can be used in those patients with resectable lesions. Radiotherapy has not been indicated for USP due to poor tumoral response.^3^, ^5^, ^6^, ^8^, ^9^

## Conclusion

UPS arising in larynx is a very rare mesenchymal malignant tumor, which more commonly affects males. Immunohistochemistry is a fundamental tool to establish the diagnosis. To the best of our knowledge, this is the third case reported of vocal cord UPS treated by frontolateral laryngectomy in the English literature.

## Conflicts of interest

The authors declare no conflicts of interest.
